# Eliciting mixed emotions: a meta-analysis comparing models, types, and measures

**DOI:** 10.3389/fpsyg.2015.00428

**Published:** 2015-04-15

**Authors:** Raul Berrios, Peter Totterdell, Stephen Kellett

**Affiliations:** ^1^Department of Psychology, University of SheffieldSheffield, UK; ^2^Departamento de Administracion, Facultad de Administracion y Economia, Universidad de Santiago de ChileChile; ^3^Sheffield Health and Social Care NHS Foundation TrustUK

**Keywords:** mixed emotions, mixed feelings, emotional complexity, affect model, meta-analysis

## Abstract

The idea that people can experience two oppositely valenced emotions has been controversial ever since early attempts to investigate the construct of mixed emotions. This meta-analysis examined the robustness with which mixed emotions have been elicited experimentally. A systematic literature search identified 63 experimental studies that instigated the experience of mixed emotions. Studies were distinguished according to the structure of the underlying affect model—dimensional or discrete—as well as according to the type of mixed emotions studied (e.g., *happy-sad, fearful-happy*, *positive-negative*). The meta-analysis using a random-effects model revealed a moderate to high effect size for the elicitation of mixed emotions (*d*_*IG*+_ = 0.77), which remained consistent regardless of the structure of the affect model, and across different types of mixed emotions. Several methodological and design moderators were tested. Studies using the minimum index (i.e., the minimum value between a pair of opposite valenced affects) resulted in smaller effect sizes, whereas subjective measures of mixed emotions increased the effect sizes. The presence of more women in the samples was also associated with larger effect sizes. The current study indicates that mixed emotions are a robust, measurable and non-artifactual experience. The results are discussed in terms of the implications for an affect system that has greater versatility and flexibility than previously thought.

Emotions are sometimes more complex than the notions we possess to communicate how we feel, above all for those affects that combine two opposite valenced emotions at the same time (i.e., mixed emotions). Mixed emotions have been defined as affective experiences characterized by the co-activation of two emotions, usually opposite in valence (Larsen et al., 2001), like for example, feeling happy and sad. Other definitions have considered mixed emotions as the intraindividual correlation between the opposite dimensions of positive affect and negative affect, where values closer to zero reflect greater emotional complexity (Grühn et al., 2013). This implies that mixed emotions are experienced over time, so that mixed emotions can be also studied as a trait. Nevertheless, in the present research mixed emotions are understood as transient feeling states that involve two opposite affects.

Affects are characterized by subjective feelings, and can be divided into two main categories: emotions and moods. Emotions are usually briefer than moods, and occur in response to a specific eliciting stimulus whereas moods are more diffuse and reflect multiple inputs. As such, mixed emotion experience can be considered to be a form of mood (Rafaeli et al., 2007), although the present work focuses on the emotions that contribute to the experience.

The feasibility of experiencing mixed emotions has aroused controversy since the beginning of the scientific psychology era. Kellogg (1915) showed that rapidly alternating the presentation of a pleasant (e.g., a cat) and an unpleasant (e.g., a surgical intervention) stimulus can instigate the experience of mixed feelings. He suggested that two opposite streams of feelings can operate continuously, unless one of these feelings is much greater in intensity, in which case, the stronger prevails. Shortly after, Young (1918) argued that such mixed feelings represent a “*meaning error*,” that is, people reporting mixed feelings confused emotions felt with emotion intellectualization, which refers to a rationalization of an event using emotion words without the corresponding feeling.

Over time, research interest has grown but controversies in the field survive. Thus, most of the research studying this complex emotional experience has been dedicated to demonstrating that mixed emotions are not a product of: demand effects, lay theories of mixed emotions, vacillation, or measurement problems (e.g., Larsen and McGraw, 2011, 2014; Schimmack, 2001, 2005; Rafaeli and Revelle, 2006; Larsen and Green, 2013). However, the extant research investigating mixed emotions has yet to systematically examine whether the variety of designs and studies performed until now consistently support the experience of mixed emotions.

Research on mixed emotions varies substantially in terms of several variables: (a) the underlying model of affect considered (i.e., dimensional or discrete emotions), (b) the type of mixed emotions studied, (c) the type of measure used to assess the presence and intensity of mixed emotions, and (d) the induction procedure used to activate mixed emotions. Hence, it is worth integrating and assessing the diverse accumulated evidence to evaluate how robust and consistent mixed emotions actually are. According to Wimsatt (1981), a robust phenomenon is one which is sufficiently invariant under a variety of conditions to reach identical conclusions about the process under scrutiny. Thus, in the present research we have assessed the robustness of mixed emotions by examining whether researchers have been equally successful in experimentally eliciting mixed emotions using different: models of affect, combinations of emotions, measures, and procedures. In this quantitative review, we use these variations to examine the robustness of mixed emotions as an affective experience.

To accomplish this goal, we first briefly review models of affect and the different types of mixed emotions that have been used to understand mixed emotions. Next we describe the methods and procedures through which researchers have tried to reveal the activation of mixed emotions. To do this we contextualize the methodological progress as reflecting an effort to support the basic assumptions behind the phenomenon of mixed emotions. Finally, we present a meta-analysis of experimental studies investigating the experience of mixed emotions and we discuss its implications for understanding mixed emotions.

## Mapping the terrain of mixed emotions: underlying models and types

We start by introducing the distinction between mixed emotions as the co-occurrence of oppositely valenced affects and emotion-blends as a category including all possible experiences combining more than one emotion (Scherer, 1998). Emotion blends have been largely studied and accepted in the emotion literature (Izard, 1972, 1992; Polivy, 1981; Folkman and Lazarus, 1985; Diener and Iran-Nejad, 1986; Smith and Ellsworth, 1987; Oatley and Johnson-Laird, 1996; Scherer, 1998). For example, people report combined feelings of both happiness and excitement or both anxiety and fear (e.g., Zelenski and Larsen, 2000; Vansteelandt et al., 2005). Thus emotion blends of similar valence are uncontentious. However, there are a number of important issues (e.g., cultural differences, emotional complexity) one of these is whether oppositely valenced affects can be experienced simultaneously (Lindquist and Barrett, 2008), which forms the focus of the present review. Views about the possibility of consciously experiencing two opposite affects depend, to an extent, on the underlying model of affect to which researchers subscribe, with the main distinction being between dimensional and basic models.

### Mixed emotions and dimensional models of affect

Dimensional models of affect propose that the best representation of the underlying structure of feelings is to locate them on dimensions. People usually describe their feelings as positive or negative, pleasant or unpleasant, which suggests that one of the fundamental dimensions is valence. Some dimensional models of affect have postulated that opposite ends of the positive-negative dimension (Russell and Carroll, 1999; Watson and Tellegen, 1999) and the underlying appetition-aversion affect systems (Grey, 1982; Lang, 1995) correspond to mutually exclusive feelings. The implication of this is that, for example, happiness and sadness cannot be experienced simultaneously, which challenges the idea that it is possible to experience mixed emotions.

In particular, scholars ascribing to the circumplex model of affect have asserted that mixed emotions are merely result of measurement problems or a reactive phenomenon related to expectancies of participants and/or researchers and arise from confusion in reports of emotion (Barrett and Bliss-Moreau, 2009). In the circumplex model, affect is represented by two orthogonal dimensions of valence and activation forming a circular space around which affect-items can be descriptively organized (Russell, 1980). The dimension of valence reflects the level of pleasantness/unpleasantness felt; whereas the dimension of activation reflects the level of arousal characteristic of the affect. Affects lying at opposite ends of each of these two bipolar dimensions are mutually exclusive, which means that an increase in high-activated pleasure implies the reduction of low-activated displeasure and vice versa. If an affective experience has a single location on these bipolar dimensions, mixed emotions are therefore an oxymoron.

In contrast, the Evaluative Space Model (ESM; Cacioppo et al., 1999, 2004) contends that affect can be characterized by a dimension of positive affect and another dimension of negative affect forming a bivariate space in which it is possible to describe multiple variations of positive and negative emotions, including mixed emotions. The ESM (Cacioppo et al., 1999, 2004) stipulates that positive affect and negative affect are biologically basic substrates of the affect system with identifiable brain structures, functionally represented throughout different levels of the neural system. This *biological architecture* allows multiple activation patterns, such as reciprocal, independent or co-activation patterns (Norris et al., 2010; Norman et al., 2011). Although physical constraints may make the affect system appear as bipolar in nature, its neurobiological architecture is better understood as bivariate (Norman et al., 2011).

### Mixed emotions and theories of basic emotion

Some theories of basic emotion also support the experience of mixed emotions. Theories of basic emotions usually consider emotional experiences as being measureable and physiologically distinct phenomena characterized by a small set of emotions (Izard, 1972). Izard (1992) argued that basic emotions can be blended to form new emotions in accordance with specific conditions occurring in the environment. Like mixing paint colors on a palette, mixed emotions result from the different possible combinations of basic emotions, such as happiness, sadness, anxiety or disgust. Izard also noted that “one emotion can almost instantaneously elicit another emotion that amplifies, attenuates, inhibits or interacts with the original emotional experience” (Izard, 1972, p. 77). Although this conceptualization is distinct from the definition of mixed emotions used in this study because it suggests the emergence of new emotions by blending basic emotions, it is interesting to note that, in this theory, emotions are freely allowed to interact regardless of valence, which suggests that mixed emotion experiences are feasible. This clarification is important because studies investigating blended emotions that form new emotions, as suggested by Izard (1992), do not fall within the focus of the present study.

More closely linked to the current understanding of mixed emotions, Oatley and Johnson-Laird (1996) proposed that individuals react to events by making multiple cognitive evaluations, which in turn, may elicit multiple basic emotions simultaneously or in rapid alternation many of which are mixed emotions, giving rise to facial expressions combining more than one basic emotion, as previously demonstrated by Ekman and O'Sullivan (1991). Recent evidence supports this assertion demonstrating that it is possible to identify 21 different and consistent facial expressions (Du et al., 2014), many of which reflect combinations of basic emotions (e.g., happily disgusted).

### Types of mixed emotions

Happy-sad has been, by large, the most common type of mixed emotion studied (e.g., Larsen et al., 2001; Williams and Aaker, 2002; Fong, 2006; Larsen and Green, 2013). For example, an emblematic study showed that students who had just moved to their dormitories felt significantly sadder but also happier than in a typical day before this event (Larsen et al., 2001, Study 2). Nevertheless, several other types of mixed emotions have been investigated, including the co-activation of fear and happiness (e.g., Andrade and Cohen, 2007), disgust and amusement (e.g., Hemenover and Schimmack, 2007), and hope and fear (e.g., Bee and Madrigal, 2013). Furthermore, different studies have conceived the experience of mixed emotions as reflecting either the co-activation of different dimensions of affect such as positive and negative affect (e.g., Henderson and Norris, 2013) and pleasure-displeasure (e.g., Schimmack and Colcombe, 2007) or as reflecting the experience of different discrete emotions such as happy-sad or hope-fear. The diversity of types of mixed emotions, including both dimensional and discrete conceptualizations, implies relevant theoretical consequences. If consistent evidence about the experience of mixed emotions is found across different models of affect, then it is possible to suggest that mixed emotions are a universal affective experience, that is, not necessarily restricted to certain “traditional” emotion combinations (e.g., happy-sad). Similarly, this diversity may suggest that the affective system is flexible enough to permit a plurality of affective experiences ranging from bipolarity to mixed emotions.

In summary, it is possible to describe mixed emotions as a multifaceted emotional experience, which involves the simultaneous experience of different combinations of opposing emotions. Both dimensional and basic emotions approaches have theorized the possibility of experiencing mixed emotions. It is important to note, however, that several studies exploring the activation of mixed emotions (e.g., Andrade and Cohen, 2007; Hemenover and Schimmack, 2007) have focused on discrete emotional experiences, thereby using a distinct model of affect, without explicitly ascribing to a basic emotion approach. An empirical examination of the consistency with which mixed emotions have been elicited for different underlying models of affect and different types of mixed emotions has not been previously conducted. This examination is needed to determine whether mixed emotions apply to the universe of emotions, or whether they are restricted to particular kinds or combinations of emotions and therefore represent a more specific phenomenon.

## Measurement and elicitation of mixed emotions

Different measures and elicitation procedures have been developed to demonstrate that opposite affects can be experienced concurrently and that mixed emotions are a genuine affective experience (Larsen and McGraw, 2014). These two assumptions—*simultaneity of opposing emotions* and the *integral experience of mixed emotions*—organize ongoing efforts to demonstrate the experience of mixed emotions. This section describes these two assumptions, explains the measures used to evaluate the presence/intensity of mixed emotions and the procedures used to elicit mixed emotions.

### The simultaneity of opposing emotions

The assumption of simultaneity is that mixed emotions reflect the co-activation of different emotions, usually described as opposite. According to the ESM (Cacioppo et al., 1999, 2004), simultaneity is achieved through two different mechanisms. Firstly, co-activation can result from perceiving both positive and negative features of a single stimulus or event (Cacioppo et al., 2011), which is supported by evidence that indicates attention can be directed to at least two steams of information (De Gelder and Vroomen, 2000). Secondly, co-activation can also result from alternations between positive and negative stimuli that are fast enough to produce sustained activation of both (Schimmack and Colcombe, 2007; Norris et al., 2010). Thus, the experience of feeling two opposite affects simultaneously may result either from the rapid alternation between emotions or the co-activation of two emotions.

Other theories of emotion have asserted that only one emotion can be activated and consciously experienced at a given time (Brehm, 1999; Russell, 2003; Brehm and Miron, 2006). For example, Brehm (1999) proposed that emotions provide guidelines for behavioral responses, consistent with the level of instrumental difficulty (i.e., deterrence) necessary to attain a certain outcome. When the deterrence is high enough a new affective response is activated, which may result in a rapid transition from one emotion into another, but they are not both consciously experienced at the same time (Brehm and Miron, 2006). However, this does not necessarily prohibit the experience of mixed emotions. As suggested by Kellogg (1915), the activation of two opposite affects in rapid succession can instigate the experience of mixed emotions until the intensity of one affect prevails, as demonstrated by Schimmack and Colcombe (2007).

### The integral experience of mixed emotions

If mixed feelings are a genuine emotional experience, they should involve distinctive states of consciousness which are experienced as personal feelings. Mixed emotions are a complex affective experience and not merely a collection of independent emotions elicited in response to separate triggers. Even though people can report identifiable environmental sources of mixed emotions, the subjective feeling reflects the co-occurrence of both positive and negative emotions. This approach assumes, therefore, that mixed emotions are more than the sum of the emotions involved; mixed feelings are in themselves a distinct and integral emotional experience.

Contrary to this assumption, Greenspan (2003) has indicated that emotions appear mixed when actually they are different emotional experiences pertaining to independent events. Thus, for example, people may report feeling happy and sad because they are feeling happy in relation to event “*A*,” and sad in relation to event “*B*.” Similarly, people can perceive and verbalize the contrasting affective qualities of external events, without experiencing any distinctive change in affective experience (Russell, 2003).

However, the subjective experience of mixed emotions is grounded in the idea that our feelings reflect two genuine affects converging upon a single reference point, that is, ourselves. For example, Hunter et al. (2008) found that conflicting musical stimuli (i.e., musical pieces in fast tempo and minor mode) created more happiness and sadness compared to non-conflicting musical pieces. This study examined mixed emotions using one (indivisible) focus of attention, avoiding alternative interpretations based on the events surrounding the emotional experience and supported the assumption that mixed emotions are an integral experience.

### Measures of mixed emotions

In order to capture the simultaneity and the subjective experience of mixed emotions, several measures have been developed. Following Hershfield and Larsen (2012), it is possible to distinguish four different measurement methods. The first corresponds to measuring the extent to which people experience positive affect, negative affect, or both together continuously (e.g., Larsen et al., 2004; Larsen and McGraw, 2011; Larsen and Green, 2013). This simultaneous measure asks participants to press a button every time they feel good and release the button when they no longer feel good and press another button whenever they feel bad and release it when they no longer feel bad. Participants can press both buttons simultaneously, when feeling good and bad, and can abstain from pressing a button if they feel neither good nor bad. This captures mixed emotions in real-time, so avoiding confounding reports due to timescale or characteristics of the scale used. This measure is usually operationalized as the amount of time participants spend pressing the two buttons simultaneously.

Secondly, based on previous works on attitudinal ambivalence (e.g., Priester and Petty, 1996), the intensity of mixed emotions has been estimated using the minimum value between positive and negative emotions. For example, if an individual reports feeling “4” for happiness on a scale from 1 to 5, and also reports feeling “2” for sadness, the minimum index of mixed emotions will be “2” (i.e., the minimum value); if one of the emotions measured (e.g., sadness) is not felt, the minimum value would be zero indicating that mixed emotion was not experienced. This index therefore reflects the intensity of the experience of mixed emotions, but it does not reflect differences in the balance between the two emotions. For example, in the same example, the index would remain at “2” even if happiness increased to its maximum value “5.” Nevertheless, a measure of mixed emotions based on a minimum value is considered more appropriate than a simple correlation between positive and negative affect because it reflects the intensity of mixed emotions (Schimmack, 2001, 2005). Other studies of mixed emotion have used similar measures based on a minimum value, such as the similarity intensity index (e.g., Williams and Aaker, 2002; Hong and Lee, 2010).

Thirdly, it is possible to infer the simultaneous experience of mixed emotions by counting the number of occasions during which people experience each emotion measured. By counting the occasions on which two or more emotions of opposite valence are experienced, it is possible to establish a raw estimation of the presence (or absence) of mixed emotions. For example, Oatley and Johnson-Laird (1996) provided evidence that people can experience mixed emotions (happiness-sadness) on almost 14% of occasions. Similar measures of mixed emotions can be constructed by evaluating the frequency of opposite emotions experienced across different groups (e.g., experimental versus control) completing an emotional questionnaire. For example, this may involve using a manipulation procedure to test whether an experimental group experiences a greater extent of two opposite emotions compared to a control group where the manipulation is not present (e.g., McGraw and Warren, 2010).

Finally, mixed emotions have been measured by asking people directly in a questionnaire whether they are experiencing mixed feelings. The specific subjective experience of emotions is an inherent part of emotional life (e.g., Helm, 2009), and self-reports of subjective feelings can be useful in this regard. For example, Berrios et al. (2015) demonstrated that subjective self-reports of mixed feelings are useful and consistent indicators of mixed emotions. Interestingly, the investigation (Study 2) produced equivalent results for subjective measures of mixed emotions and the minimum index.

### Elicitation of mixed emotions

Several procedures have been developed to elicit mixed feelings. Common emotion induction procedures have been films (e.g., Larsen et al., 2001), music (e.g., Hunter et al., 2008), pictures (e.g., Schimmack and Colcombe, 2007), and advertisements (Andrade and Cohen, 2007). For example, in one study Larsen and McGraw (2011, Study 1a) demonstrated that people experienced both happiness and sadness whilst watching a film-clip from the movie *Life is Beautiful*. In recent years more ecologically valid techniques such as recalling personal experiences in response to conflicting goals (e.g., Berrios et al., 2015), have been employed to expand understanding of the precursors of mixed emotions.

In summary, different elicitation procedures have been developed and tested to investigate the presence and intensity of mixed emotions. Moreover, multiple measures have been developed to support both the simultaneity and the integral experience of mixed emotions. From the measures described, it is possible to suggest that the simultaneous measure of mixed emotions would have more consistent effect sizes compared to other measures in identifying the co-activation of opposite affects because it overcomes problems arising from the timescale and the measurement scale used. Additionally, subjective measures of mixed emotions rely more on the second assumption, according to which mixed emotions can be both mentally represented and experienced. The diversity of measures and procedures used to study mixed feelings has yet to be integrated in terms of their relative influence in capturing and characterizing the experience of mixed emotions.

## The present research

The present research examines and quantifies the robustness of mixed emotions by meta-analyzing extant research. If mixed emotions are a robust phenomenon, they should be consistent over and above any artifactual variable (such as measurement error or design characteristic) and across different theoretical or methodological characteristics of the studies included. This meta-analysis investigated the effects of: (a) distinguishing between dimensional and discrete emotions approaches to the study of mixed feelings, and (b) the type of mixed emotions tested. Differences arising from separate models and different types of mixed emotions therefore enable conclusions to be drawn about the degree of generalizability and specificity of mixed feelings. Several methodological characteristics were included as moderators to determine their impact in the elicitation of mixed emotions, including: (a) measures used, (b) induction procedures (c) design characteristics, and (d) demographic characteristics. The measures used are of particular value as they reflect different assumptions about mixed emotions (i.e., simultaneity of opposing emotions and the integral experience of mixed emotions), so evidence for the validity of these assumptions can be gleaned from the relative effect size produced. Induction procedures and design characteristics (i.e., within or between person designs) were included because they help rule out the possibility that mixed emotions are artifacts of study design, rather than a genuine emotional experience. Finally, demographic characteristics were included to enable future research to focus on promising samples. Specifically, age and gender were studied because several studies have found a positive association between age and experiencing mixed emotions (e.g., Carstensen et al., 2000; Ong and Bergeman, 2004), while other studies have shown variations in the experience of mixed emotions as a function of gender (e.g., Larsen et al., 2001; Berrios et al., 2015).

## Method

### Selection of studies

The sample of studies used in the meta-analysis was obtained by conducting a computerized search (via Web of Knowledge, and PsycINFO, Dissertation Abstracts International) for articles published before January, 2014, using the keywords: “*mixed emotions*,” “*mixed feelings*,” “*emotional blends*,” “*emotional ambivalence*,” “*contrasting emotions*,” or “*emotional complexity*.” Articles had to include the respective terms either in the title, abstract or keywords. Reference lists in some articles were inspected to identify additional sources for inclusion. Furthermore, emails to relevant researchers in the field were sent in order to incorporate potential unpublished studies. Similarly, a public advertisement was placed on *ResearchGate* (an international online social network for researchers) inviting researchers to share any unpublished studies investigating mixed emotions. The literature search identified 826 articles and dissertations.

Four inclusion criteria were considered for the meta-analysis. First, studies had to employ an experimental design and recruit a human, non-clinical sample. Experiments were chosen because: (a) they provide a meaningful counterfactual condition(s) against which to compare the activation of mixed emotions, and (b) the allocation of participants is random—or at least quasi-random—enhancing the adequate interpretation of the effect sizes. Experiments based on comparisons between cultures were only included if the samples contained participants from different cultural backgrounds randomly allocated to the experimental and control condition(s).

Second, studies had to manipulate the experience of mixed emotions using films, images, music, or any other procedure that was deemed by the studies to instigate the experience of mixed emotions. Importantly, studies had to manipulate mixed emotions and report the effectiveness of this manipulation on participants' emotional experience in comparison with emotional experience in specific control condition(s) (i.e., between-participant designs) and/or in comparison with participants' emotional experience before the corresponding manipulation (between-within-participant designs).

Third, studies had to measure mixed emotions, that is, studies needed to consider the experience of two opposite affects as co-occurring; studies in which other emotional blends were measured were not included (e.g., anxious-fear). The classification of mixed emotions was based on the hedonic valence of the emotions involved. Thus studies were included if they tested a positively and a negatively valenced emotion. Similarly, if the study incorporated a dimensional approach, the following combinations were included; positive-negative affect, positive activated-negative activated affect, pleasant-unpleasant affect.

Fourth, studies had to report a measure of mixed emotions that reflected the magnitude of the mixed emotion experienced (for example, by using the common indices of mixed emotions described in the introduction). Correlational indices were not considered unless two or more correlations were compared between experimental and control conditions (e.g., Andrade and Cohen, 2007, Study 3a). General correlational indices were not considered, as correlations are not an appropriate measure for the experience of mixed emotions (Schimmack, 2001).

Of the 826 articles and theses identified by the search, 47 articles met the inclusion criteria from which we were able to compute effect sizes for 35 articles involving a total of 63 independent studies. The other 12 articles were excluded because we could neither compute precise effect sizes nor estimate effects in the studies reported; authors were contacted where possible in an attempt to include these data. Each of the selected articles is identified by an asterisk in the reference list. We computed precise effect sizes for 60 studies (95%) on the basis of information in the article; whereas for 3 studies (5%), we had to estimate some or all values based on the significance levels reported.

### Selection of comparisons within studies and selection of moderators

Two types of comparisons were examined in order to provide information about the relevance of different models for investigating mixed emotions, and the nature of mixed emotions. First, a comparison between dimensional models of affect and discrete emotions approaches was performed; studies considering dimensions of affect (e.g., positive-negative affect) were compared with studies in which discrete emotions were measured (e.g., happy-sad). Second, we compared different types of mixed emotions, that is, happy-sad, fear-happy, disgust-amusement, hope-fear, positive-negative affect, and pleasant-unpleasant affect. Such comparisons help to clarify the generalizability and diversity of mixed emotions.

To evaluate the effect of the moderator variables, studies were coded according to two methodological factors and three study characteristics. Firstly, the measure of mixed emotions used was coded based on a 4-fold classification: (i) simultaneous measures of mixed emotions, (ii) measures of mixed emotions using the minimum index or derivations of a similar formula, such as the similarity-intensity index (e.g., Williams and Aaker, 2002), (iii) measures of mixed emotions based on the frequency of opposing affects, and (iv) subjective measures of mixed emotions. Studies reporting a combination of measures were coded as a “mix of measures,” and the effect sizes obtained from each measure were averaged. Secondly, the emotion induction procedure was coded according to whether ads, films, music, pictures, personal experiences, simulation or imagination (e.g., participants imagine a situation or remember a recent event), or another—unclassified—induction procedure was used. Thirdly, the design characteristic of each study was coded according to whether it used a within-person or between-person design. Finally, two demographic characteristics—mean age and percentage of women—were coded. Further details about the study characteristics and the effect sizes for each study are provided in Table 1.

**Table 1:** **Characteristics and effect sizes for studies included in the meta-analysis**.

**Study**	**Experiment**	**Type of mixed emotion**	**Mixed emotion indicator**	**Induction procedure**	***N*_*E*_**	***N*_*C*_**	**Effect size**
Aaker et al., 2008	2	Positive, negative	MIN	Ads	45		1.01
Andrade and Cohen, 2007	2	Fear, happy	SIM	Other	75		0.58
Andrade and Cohen, 2007	3a	Fear, happy	SIM	Other	81		2.12
Barrett et al., 2010	1	Positive, negative	FRQ	Music	226		0.52
Bee and Madrigal, 2013	1	Hope, fear	MIN	Ads	54	106	0.41
Bee and Madrigal, 2013	2	Hope, fear	MIN	Ads	41	80	0.71
Berrios et al., 2015	1	Positive, negative	SUB	Personal experiences	22	13	0.84
Berrios et al., 2015	2	Positive, negative	MIX	Personal experiences	30	27	0.87
Carrera and Oceja, 2007	2	Happy, sad	FRQ	Pictures	37	39	0.94
Fong, 2006	1	Happy, sad	MIX	Personal experiences	27	75	0.79
Fong, 2006	2	Happy, sad	MIX	Film	74	64	0.94
Fong and Tiedens, 2002	1	Positive, negative	MIN	Simulation	27	25	0.59
Hemenover and Schimmack, 2007	1	Disgust, amusement	MIN	Other	49	53	0.48
Henderson and Norris, 2013	1	Positive, negative	MIN	Simulation	30		1.76
Hershfield et al., 2009	1	Positive, negative	MIN	Simulation	22	23	1.37
Hershfield et al., 2008	1	Happy, sad	MIN	Simulation	60	60	0.73
Hershfield et al., 2008	2	Happy, sad	MIN	Simulation	51	59	0.45
Hong and Lee, 2010	1	Positive, negative	MIN	Pictures	45	46	0.60
Hong and Lee, 2010	2	Positive, negative	MIN	Pictures	37	38	0.59
Hong and Lee, 2010	3	Positive, negative	MIN	Pictures	125	125	0.43
Hong and Lee, 2010	4	Positive, negative	MIN	Pictures	74	75	0.20
Hong and Lee, 2010	5	Positive, negative	MIN	Pictures	108	109	0.78
Hunter et al., 2008	1	Happy, sad	MIN	Music	40		0.70
Hunter et al., 2008	2	Happy, sad	MIN	Music	40		1.13
Hunter et al., 2010	1	Happy, sad	MIN	Music	49		0.72
Kreibig et al., 2013	1	Disgust, amusement	MIN	Film	43		2.50
Ladinig and Schellenberg, 2012	1	Happy, sad	FRQ	Music	61		1.20
Larsen and Green, 2013	1	Happy, sad	SIM	Film	40		2.39
Larsen and Green, 2013	2	Happy, sad	SIM	Film	55		0.17
Larsen et al., 2009	1a	Happy, sad	SIM	Film	22	25	0.18
Larsen et al., 2009	1b	Happy, sad	MIN	Film	17	22	0.19
Larsen et al., 2009	2	Happy, sad	FRQ	Film	21	18	0.23
Larsen et al., 2009	3	Happy, sad	SIM	Film	28	24	0.14
Larsen et al., 2009	4	Happy, sad	MIX	Film	33	83	0.95
Larsen et al., 2009	5	Happy, sad	MIX	Film	33	61	0.88
Larsen et al., 2009	6	Happy, sad	MIX	Film	50	24	0.73
Larsen et al., 2001	1	Happy, sad	FRQ	Film	177	177	0.88
Larsen et al., 2001	2	Happy, sad	FRQ	Personal experiences	100	92	0.78
Larsen et al., 2001	3	Happy, sad	FRQ	Personal experiences	115		0.72
Larsen et al., 2004	1	Positive, negative	MIN	Simulation	20		0.86
Larsen et al., 2004	2	Positive, negative	SIM	Simulation	20		0.76
Larsen et al., 2009	2	Positive, negative	MIN	Personal experiences	19		0.97
Larsen and Stastny, 2011	1	Happy, sad	SIM	Music	21		0.59
Madrigal and Bee, 2005	1	Hope, fear	MIN	Ads	36		1.23
McGraw and Warren, 2010	3	Disgust, amusement	FRQ	Simulation	36		0.73
McGraw and Warren, 2010	4	Disgust, amusement	FRQ	Simulation	80		0.81
McGraw and Warren, 2010	5	Disgust, amusement	FRQ	Simulation	73		1.27
Oceja and Carrera, 2009	1	Positive, negative	MIN	Pictures	37	69	0.98
Oceja and Carrera, 2009	2	Positive, negative	MIN	Pictures	61	29	1.05
Rees et al., 2013	3a	Happy, sad	SUB	Simulation	53		1.80
Rees et al., 2013	3b	Happy, sad	SUB	Simulation	652		1.56
Schimmack and Colcombe, 1999	1	Pleasure, displeasure	FRQ	Pictures	36		0.76
Schimmack and Colcombe, 1999	2	Pleasure, displeasure	MIN	Pictures	44		0.67
Schimmack, 2001	1	Pleasure, displeasure	MIN	Pictures	342		0.76
Schimmack, 2005	1	Pleasure, displeasure	MIN	Pictures	1118		0.39
Schimmack and Colcombe, 2007	1	Pleasure, displeasure	MIN	Pictures	80		0.50
Spencer-Rodgers et al., 2010	1	Positive, negative	MIN	Other	54	53	0.46
Veilleux et al., 2013	1	Positive, negative	MIN	Pictures	100		0.67
Williams and Aaker, 2002	1	Happy, sad	MIN	Ads	204		0.52
Williams and Aaker, 2002	2	Happy, sad	MIN	Ads	59	70	0.30
Williams and Aaker, 2002	3	Happy, sad	MIN	Ads	88		0.61
Zhang et al., 2010	1	Happy, sad	MIN	Simulation	30		0.76
Zhang et al., 2010	2	Happy, sad	MIN	Simulation	58		0.64

N_E_, number of participants in the experimental condition; N_C_, number of participants in the control condition/s; MIN, minimum index or similar indicator; SIM, simultaneous measure of mixed emotions; FRQ, indicator of mixed emotions based on frequencies; SUB, subjective measure of mixed emotions; MIX, multiple indicators of mixed emotions (implies studies reporting more than one measure of mixed emotions).

### Calculation of effect sizes

We calculated effect sizes that represented the degree to which mixed emotions were elicited following the affect-manipulation procedure used in each study. Thus the presence of mixed emotions was represented by a positive effect size; whereas the absence of mixed emotions was represented by values close to zero. Although it seemed unlikely, more mixed emotions among the control, relative to the experimental, condition (or before, relative to after, the affect induction) would be indicated by a negative effect size. The inclusion of both between-person and within-person study designs meant that we had to analyze data from different experimental designs. We therefore adopted Morris and DeShon's (2002) method for combining results across independent-groups and repeated measures designs. As the research question concerned the robustness and consistency in the activation of mixed emotions across different theoretical and methodological distinctions, all effect sizes were transformed into a common independent-groups metric (*d*_*IG*_) following formulations and procedures indicated by Morris and DeShon (2002). Transforming effect sizes into alternate metrics requires an estimate of the population correlation between pre- and post-test scores (Morris and DeShon, 2002). This is a common procedure to correct for measurement error which has been viewed as a typical study artifact in meta-analysis (Schmidt, 2010). We calculated this estimate using data from the strongest study available (Schimmack, 2005) in terms of the sample size, encompassing 1118 participants (16% of the total summed sample), and then we corrected for this estimate (ρ = 0.35) in the rest of the studies.

### Meta-analytic strategy

Computations were undertaken using SPSS macros designed by Wilson (2005). Weighted average effect sizes (*d*_*IG*+_) were based on a random effects model due to the assumption that the true effect sizes may vary as a function of the different models of affect and types of mixed emotions reported and as a function of the characteristics of the population (i.e., proportion of women and men; ages of the participants within samples; samples from different countries). The restricted maximum likelihood (REML) method was used to calculate the effect sizes, as it estimates more conservative standard errors (Raudenbush, 1994) and REML is more sensitive with small sample sizes (Thompson and Sharp, 1999). Effect sizes were interpreted using Cohen's (1992) guidelines which suggest that, *d* = 0.20 should be considered a “small” effect size, *d* = 0.50 is a “medium” effect size, and *d* = 0.80 is a “large” effect size. These categories were considered appropriate to the current context because the largest contemporary meta-analysis of the elicitation of emotions in general (Lench et al., 2011) has reported an average effect size of 0.51.

The homogeneity *Q* statistic (Cochran, 1954) was used to evaluate the variability in effect sizes from the primary studies. *Q* is a diagnostic tool that can be used to determine whether there is unexplained variability in the studies selected (Shadish and Haddock, 1994). Homogeneity is rejected when the *Q* statistic is significant. The homogeneity *Q* statistic was also used to compare effect sizes between different models of affect and different types of mixed emotions. We used the METAF macro for SPSS (Wilson, 2005) to estimate differences between models of affect and types of mixed emotions. This macro performs the analog to One-Way ANOVA analysis and is suitable for estimating random effects models. Similarly, the METAREG macro for SPSS (Wilson, 2005) was used to conduct meta-regressions to evaluate 16 potential moderators of effectiveness in the elicitation of mixed emotions.

## Results

### The magnitude of mixed emotions as a phenomenon

**Figure 1:**
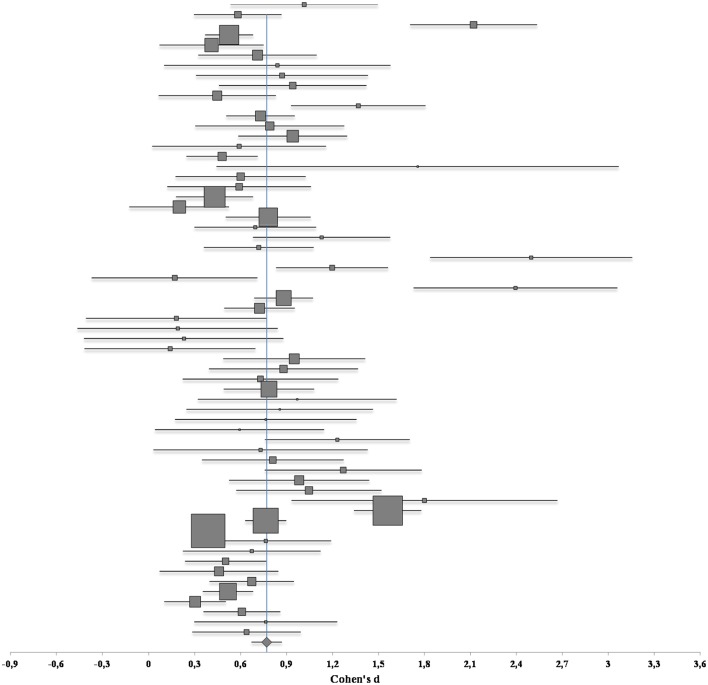
**Forest plot of effect sizes from included studies incorporating 95% CI.** Note: The presentation of studies follows the alphabetic order displayed in Table 1.

To determine the magnitude of elicited mixed emotions we calculated the sample-weighted average effect size from the primary studies. The result showed a significant medium to large average effect size, *d*_*IG*+_ = 0.77, *z* = 15.82, *p* < 0.01, with a 95% confidence interval lying between 0.68 and 0.87, based on 63 studies and a total sample size of 7157 participants. The classification of this overall effect size is also medium to large considering a recent very large (*k* = 687) meta-analysis on the elicitation of emotional experiences (*g* = 0.51; Lench et al., 2011). This indicates that mixed emotions were of sufficient magnitude to be reliably detected under a variety of conditions. The homogeneity statistic demonstrated the presence of unexplained heterogeneity, *Q*_(62)_ = 341.11, *p* < 0.01, *v* = 0.10, which confirmed the pertinence of a random effects model for the present meta-analysis. The corresponding forest plot including all the studies and the weighted average effect size are shown in Figure 1; studies with larger sample sizes are represented using proportionally bigger square symbols, and the diamond symbol reflects the weighted average effect size. In general, studies with larger sample sizes (and consequently higher power) were closer to the weighted average effect size estimated, and only a small portion of studies—commonly those with the smallest sample sizes—diverged largely from the average effect size.

### Comparing the experience of mixed emotion between models of affect and between different types of mixed emotions

There was no significant difference between studies that conceptualized mixed emotions using a dimensional or a discrete structure of affect, *Q*_(1)_ = 0.83, *n.s.* (see Table 2). The average effect size of mixed emotions measured using dimensions of affect was medium to large, *d*_*IG*+_ = 0.71, *z* = 8.28, *p* < 0.01, with a 95% confidence interval from 0.54 to 0.88, based on 24 comparisons and a total sample size of 3339. Mixed emotions measured using a discrete emotions approach showed a medium to large average effect size, *d*_*IG*+_ = 0.81, *z* = 12.14, *p* < 0.01, with a 95% confidence interval from 0.68 to 0.94, based on 39 comparisons and a total sample size of 3818. This means that dimensional and discrete emotions approaches produce similar accounts for the magnitude of experience of mixed emotions.

**Table 2:** **Average effect sizes across models of affect and across different types of mixed emotions**.

**Variable**	***d*_*IG*+_**	***SE***	***K***	***I*^2^**
**MODEL OF AFFECT**				
Dimensional	0.71^**^	0.09	24	0%
Discrete	0.81^**^	0.07	39	34.9%^a^
**TYPE OF MIXED EMOTIONS CONSIDERED**				
Happy-sad	0.77^**^	0.07	29	17.1%
Fear-happy	1.28^**^	0.27	2	87.3%^b^
Disgust-amusement	1.07^**^	0.19	5	68.0%^a^
Hope-fear	0.53^*^	0.22	3	0%
Positive-negative	0.75^**^	0.09	19	0%
Pleasure-displeasure	0.60^**^	0.17	5	0%

SE and k are standard error and number of studies, respectively. I^2^ is a quantification of the degree of heterogeneity calculated by I^2^ = 100% × (Q–df)/Q, where Q is Cochran's heterogeneity statistic and df the degrees of freedom (Higgins et al., 2003).

** p < 0.01;

* p < 0.05.

a Q_w_ significant at p < 0.05;

b Q_w_ significant at p < 0.01.

Furthermore, no significant difference were found between the six types of mixed emotions measured in the primary studies, *Q*_(5)_ = 8.06, *n.s.* Considering first the two types of mixed emotions included within the dimensional approach, the average effect size for a mixed emotion formed from the combination of *positive-negative* was medium to large, *d*_*IG*+_ = 0.75, 95% *CI* = 0.58–0.94. A medium effect size was observed for the pair *pleasure-displeasure*, *d*_*IG*+_ = 0.61, 95% *CI* = 0.28–0.93. A comparison between the two pairs of mixed emotions within the dimensional model of affect yielded no significant difference, *Q*_(1)_ = 0.99, *n.s.*

Turning now to the discrete mixed emotion pairs, there was a medium to large effect size for the mixed emotion combination *happy-sad*, *d*_*IG*+_ = 0.77, 95% *CI* = 0.62–0.92. A large effect size was also found for the mix *fear-happy*, *d*_*IG*+_ = 1.28, 95% *CI* = 0.74–1.82, and *disgust-amused*, *d*_*IG*+_ = 1.07, 95% *CI* = 0.69–1.44. Finally, a medium effect size was found for the mixed emotion combination of *hope-fear*, *d*_*IG*+_ = 0.53, 95% *CI* = 0.08–0.97. These results indicate that different combinations of mixed emotions all produced substantial effects.

Comparisons across the discrete pairs of mixed emotions showed no significant differences between any of the pairs: *happy-sad* and *fear-happy*, *Q*_(1)_ = 2.63, *n.s.*; *happy-sad* and *hope-fear*, *Q*_(1)_ = 1.07, *n.s.*; *happy-sad* and *disgust-amused*, *Q*_(1)_ = 1.85, *p* > 0.10; *fear-happy* and *hope-fear*, *Q*_(1)_ = 1.93, *n.s.*; *fear-happy* and *disgust-amused*, *Q*_(1)_ = 0.10, *p* > 0.10; *hope-fear* and *disgust-amused*, *Q*_(1)_ = 1.94, *n.s.* It should be noted, however, that high unexplained heterogeneity was found for the mixed emotions *fear-happy*, *Q*_*w*(1)_ = 7.90, *p* < 0.01, and *disgust-amusement*, *Q*_*w*(4)_ = 12.50, *p* < 0.05.

### Moderators of the effectiveness of mixed emotions elicitation

Several methodological factors and study characteristics were tested as potential moderators of the effectiveness with which mixed emotions were elicited (see Table 3). First, the type of measure used to evaluate mixed emotions was tested. Studies including the minimum index reported smaller effect sizes on average compared to studies not using this measure, β = −0.20, *z* = −1.97, *p* = 0.04. In contrast, direct measures of mixed emotions reported marginally larger effect sizes than studies not including this measure, β = 0.57, *z* = 1.92, *p* = 0.05. The use of simultaneous measures and frequency-based measures did not influence effect sizes.

**Table 3:** **Moderators of the effectiveness of mixed emotions elicitation**.

**Moderator**	**Regression coefficient**	**Standard error**	***k***	***n***	**95% *CI***	***I*^2^**
**INDICATOR OF MIXED EMOTIONS (ABSENT, PRESENT)**						
Minimum index	−0.20^*^	0.10	28/35	2823/4334	−0.40/−0.01	0%
Simultaneous measure	0.10	0.17	55/8	6766/391	−0.23/0.43	77.2%^b^
Frequency	0.04	0.14	52/13	6046/1111	−0.23/0.31	0%
Subjective measure	0.57^†^	0.30	60/3	6417/740	−0.03/1.12	16.7%
Mix of measures	0.10	0.19	57/6	6576/581	−0.27/0.46	0%
**MIXED EMOTIONS INDUCTION PROCEDURE (ABSENT, PRESENT)**						
Ads	−0.25	0.16	56/7	6374/783	−0.55/0.06	0%
Films	0.07	0.14	51/12	6243/914	−0.21/0.35	66.3%^b^
Music	0.03	0.17	57/6	6720/437	−0.32/0.38	0%
Pictures	−0.16	0.12	49/14	4383/2774	−0.40/0.07	0%
Personal experiences	0.04	0.19	57/6	6637/520	−0.33/0.40	0%
Simulation or imagination	0.18	0.13	48/15	5686/1471	−0.07/0.43	0%
Other	0.24	0.23	60/3	6899/258	−0.22/0.69	81.8%^b^
**DESIGN CHARACTERISTICS**						
Within-person design	0.13	0.10	32	3619	−0.08/0.38	30.6%
Between-person design	−0.13	0.11	31	3583	−0.34/0.08	0%
**DEMOGRAPHIC CHARACTERISTICS**						
Percentage of women (range 0%– 100%)	0.89^**^	0.25	46	5097	0.20/1.58	19.6%
Age (range 18–47 years)	−0.01	0.01	27	3153	−0.02/0.02	10.2%

Columns k and n represent number of studies and number of participants, respectively. Where applicable, these are reported separately for each level of the moderator variable (indicated in parentheses at the end of each moderator name). Where a variable is coded as “absent, present,” absent was coded as 0 and present was coded as 1; thus, a positive regression coefficient indicates that studies in which the variable was present had larger effect sizes, and a negative regression coefficient indicates that studies where that variable was present had smaller effect sizes.

** p < 0.01;

* p < 0.05;

† p < 0.10.

b , Q_w_ significant at *p* < 0.01.

In terms of the procedure used to induce mixed emotions, none of the induction procedures influenced effect sizes (See Table 3). Similarly, the type of experimental design, that is, within-person designs or between-person designs, did not influence effect sizes. Finally, considering demographics characteristics, the magnitude of the mixed emotions effect was greater when there was a higher percentage of women in the sample, β = 0.89, *z* = 2.56, *p* = 0.01. Age did not influence the experience of mixed emotions.

### Publication biases

**Figure 2:**
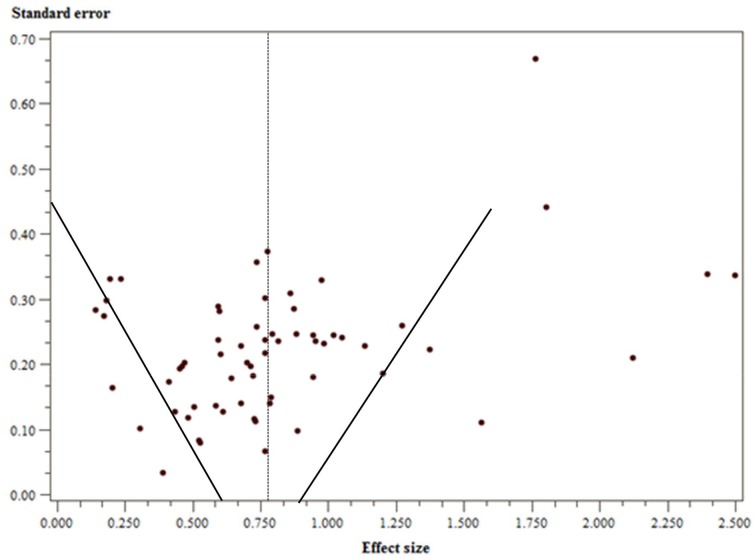
**Funnel plot of effect sizes from included studies**.

To determine whether the estimated effect sizes were biased because of missing unpublished manuscripts with small or non-significant effects, the distribution of the effect sizes observed in the primary studies was examined using a funnel plot (see Figure 2). The funnel plot showed some signs of asymmetry; some studies with small standard errors were skewed toward a positive effect, larger than the average effect size estimated. However, statistical methods to detect publication bias did not provide sufficient evidence that this bias was severe in the sample of studies. The Begg's Rank Correlation method (Begg and Mazumdar, 1994) did not show a significant presence of bias, *tau* = 0.14, *z* = −1.284, *p* = 0.10. Furthermore, contemporary methods to detect publication bias using a conditional estimator (PET-PEESE; Stanley and Doucouliagos, 2014) did not reveal the presence of severe distortions in the effect sizes (see Table 4). Despite evidence of funnel plot asymmetry demonstrated by the coefficients in the regression models (i.e., β_1_), results from applying PET-PEESE indicated that the null hypothesis that β_0_ = 0 using PET should be rejected and consequently the intercept from PEESE should be used as the best estimate of the true effect size. This was true considering both the full sample and separate samples based on the distinction between dimensional and discrete approaches.

**Table 4:** **Results from PET-PEESE indicator based on Stanley and Doucouliagos (2014) approximation to reduce publication selection bias**.

**Sample**	**PET**		**PEESE**	
	**β_0_**	**β_1_**	**β_0_**	**β_1_**
Full	0.41^**^ (0.27, 0.54)	1.86^**^	0.53^**^ (0.43, 0.63)	4.66^**^
**MODEL OF AFFECT**				
Dimensional	0.38^**^ (0.27, 0.48)	1.72^**^	0.47^**^ (0.38, 0.55)	4.38^**^
Discrete	0.55^**^ (0.21, 0.89)	1.28	0.66^**^ (0.46, 0.86)	3.26

Full, the full sample. For PET and PEESE, β_0_, the intercept (i.e., the corrected estimate of the overall effect); β_1_, the coefficient for standard error or variance (i.e., the test for funnel plot asymmetry). Numbers given in parentheses are the lower and upper limits of the 95% confidence intervals.

** p < 0.01.

Finally, a fail-safe N test for a meta-analytic random-effects model (Rosenberg, 2005) determined that 167 unpublished studies with zero effect size would have to exist in order to overturn the finding that mixed emotions are consistently elicited across the primary studies. The fail-safe N test for a fixed-effects model, similar to Rosenthal's fail-safe N (1979), determined that more than 10,000 studies would be needed to overturn the current findings; this number exceeds the suggested tolerance value of 5*n* + 10 (where *n* is the number of studies). Overall, the data appear to be resilient to publication bias.

## Discussion

This meta-analytic review examined the robustness with which mixed emotions have been elicited across a variety of theoretical and methodological contexts. Robustness was understood as the stability of effect sizes across a variety of theoretical and methodological conditions (Wimsatt, 1981). An assessment of robustness is a desirable goal for any model of affect in order to determine that accrued evidence is not a result of: methodological artifacts, the selection of certain measures, emotional adjectives, or chance. In relation to emotion theory, this evidence also contributes toward the construction of a more comprehensive theory of affect.

Accurate interpretation of research findings can be hampered by the influence of design artifacts. Schmidt (2010) stated that an effective method of avoiding this problem in meta-analysis is to use a random effects model so as to not leave significant variability unaccounted for. Additionally, Schmidt urged researchers to correct for the biasing effects of measurement errors when conducting meta-analysis. In line with these recommendations, the current research used a random effects model and corrected for measurement error using the strongest study available, enhancing confidence that the elicitation of mixed emotions appears a non-artifactual emotional experience.

Numerous studies have investigated mixed emotions, especially following several articles at the turn of the millennia that debated the structure of affect and the possibility of experiencing two opposite affects concurrently (e.g., Russell and Carroll, 1999). The present meta-analytic review assessed the evidence available and found that elicitation of mixed feelings is a robust effect. The average effect size observed from *k* = 63 experimental studies was medium to high, and the effect-sizes appeared to be resilient to publication bias. Furthermore, effect sizes were of similar magnitude regardless of whether the structure of affect was considered as dimensional or discrete and no significant difference was found when comparing effects from the different models. These results might reflect something specific to the emotions that have been studied, and in consequence, should not be extrapolated to other emotion research domains where the use of dimensional and discrete models of affect might be still contentious. However, the findings do indicate that it is unlikely that mixed emotions represent a peculiarity of certain opposing emotions (e.g., happy-sad).

Furthermore, the present review found that use of the minimum index of mixed emotions was associated with smaller effect sizes, whereas subjective measures of mixed emotions produced marginally greater effect sizes. It is possible that the minimum index is a more conservative indicator of the presence of mixed emotions because it reflects the lower threshold in the experience of mixed emotions rather than the intensity of the overall experience. In contrast, subjective measures may be more liberal in their estimation of mixed emotions because they rely on self-reports of the direct experience of mixed emotions and thereby encapsulate the integral experience of mixed emotions. The larger effect size for subjective measures provides preliminary support for the assumption that mixed emotions are an integral experience. However, it is important to mention that all the mixed emotions measures considered in the present meta-analysis may suffer from memory biases (Kihlstrom et al., 2000), except the simultaneous measure of mixed emotions.

One final finding of note is that gender moderated the elicitation of mixed emotions. Specifically, the present findings suggest that, compared to men, women either: (a) tend to experience more intense mixed emotions in response to induction procedures, (b) are more aware of experiencing mixed emotions, or (c) are more inclined to report the experience. Fujita et al. (1991) reconciled previous research showing that women report as much happiness as men but simultaneously also report greater unpleasant affect, demonstrating that women experience more intense positive emotions that balances any negative bias. Our results may offer an alternative explanation that is based on a gender difference in the experience or reporting of mixed emotions that is not effectively or adequately captured by the most commonly used affect measures.

### Implications for models of affect

Despite notable divergences in the understanding of emotion between dimensional and discrete models of affect, our findings demonstrate consistent evidence of the activation of mixed emotions using either dimensional (e.g., positive-negative affect) or discrete emotion (e.g., happy, sad) concepts. However, it is less clear under which circumstances the affect system follows a bipolar relation between positive and negative affect or activates complex affective experiences, such as mixed emotions. The evidence presented here suggests that the critical question concerning the structure of affect may not be how singular feelings relate, but rather what conditions promote different activation patterns of affect? This question is consistent with propositions found in a number of emotion theories emphasizing the role of situations in the conceptualization of affective experience (e.g., Arnold, 1960; Zajonc, 1984; Parkinson, 1997; Clore and Ortony, 2013).

The consistent occurrence of mixed emotions points to an affect system that is flexible enough to spontaneously permit multiple activation patterns, ranging from bipolar affect reactions to different blends of mixed emotions. This claim is supported by recent evidence showing that mixed emotions involve particular physiological responses that are not simply reducible to their constituent emotions (e.g., Henderson and Norris, 2013; Kreibig et al., 2013), and is also consistent with some models of affect reviewed here (e.g., Oatley and Johnson-Laird, 1996; Cacioppo et al., 2004).

Although recent findings in the field have made considerable progresses in demonstrating that genuine co-activation could be possible (e.g., Larsen and McGraw, 2011; Larsen and Green, 2013), one pending question in the field is whether mixed emotions reflect the genuine co-activation of two opposite-affects or merely the rapid succession between them. If propositions derived from the ESM are correct, both co-activation and rapid succession of affect may result in experiencing mixed emotions (Norris et al., 2010; Cacioppo et al., 2011). We suggest that conceptualizing mixed emotions as an integral distinctive experience that is more than the sum of its constituent emotions may contribute to explain how different mechanisms produce mixed emotions. The evidence presented here shows that the results for subjective measures of mixed emotions are consistent with, but not the same as, those for other types of mixed emotions measures. This offers some support to the notion of mixed emotions as an integral experience, but as few studies have used this type of measure to date such a conclusion may be premature and warrants further examination.

### Limitations and future directions

In accordance with the aims of the research, the current meta-analysis was based exclusively on studies involving experimental designs in order to provide controlled conditions to compare the experience of mixed emotions. This limitation implies that it is not possible to infer mixed emotions at the trait level, and in consequence future studies should explore the robustness of mixed emotions using ecologically valid methods, such as diary studies. Future advances in understanding the functionality of mixed emotions will also require the use of ecologically valid methods. Several diary studies have explored the within-person association between positive and negative affect (e.g., Diener and Iran-Nejad, 1986; Ong and Bergeman, 2004), so it would be interesting to analyze these and additional studies of the same kind to determine the natural conditions under which people tend to feel more mixed emotions. For example, previous studies have shown that some people tend to experience more mixed emotions under stressful situations, and that these emotional experiences can provide a buffer against the negative impact of stress in their lives (Reich et al., 2003; Davis et al., 2004). This evidence might illuminate the investigation of the contextual conditions that elicit mixed emotions and also exploring efficacy of interventions that aim to produce change in mixed emotions.

Additionally, it is worth noting that the present review investigated the hypothesis that mixed emotions are consistently activated across different theoretical and methodological conditions. This involved testing the null hypothesis that following an induction people did not experience more mixed emotions than a control group, leading to effect sizes close to zero. However, in line with assumptions derived from the ESM which suggests that the affect system can operate in multiple modes of activation (Cacioppo et al., 1999, 2004), future studies should investigate under which circumstances the affect system tends to activate one affect exclusively or two affects concurrently.

Another limitation of the present meta-analytic review is that it has only considered studies that have explicitly investigated the activation of mixed emotions, and not other studies in which positive and negative affect have been measured. Theoretically, it would be possible to calculate a mixed emotions index for those studies, but in practice reported values typically refer to average scores from which it is not possible to infer an appropriate estimation of mixed emotions. Future emotion research would be enhanced by researchers heeding the potential presence of mixed emotions when measuring emotion.

We also hope that the present results concerning mixed emotions will motivate researchers to explore whether the presence of mixed emotions can expand our understanding of common emotion-related outcomes, such as well-being and behavior. Both the ESM (Cacioppo et al., 1999, 2004) and the communicative model of emotion (Oatley and Johnson-Laird, 1996) anticipate that one consequence of experiencing mixed emotions is that they enable disparate courses of action to be followed. In addition, we suggest that the experience of mixed emotions may be important in assisting behavioral equilibration and sense-making processes. Following Piaget's conceptualizations, Bless and Fiedler (2006) have suggested that positive affect is related to *assimilative*, heuristic processing styles; whereas negative affect has been linked to *accommodative*, analytical processing styles. Using the same Piagetian analogy, we propose that mixed emotions are more likely to be associated with *equilibration* which is the process through which complex information is incorporated to restore behavioral control. Hence, mixed emotions may be the hallmark of a versatile affect system that provides advantages in flexibly interpreting a complex environment and learning to deliver adaptive responses, but that hallmark has yet to be established.

## Conclusion

The current meta-analysis has made four distinct contributions to the study of mixed emotions. First, it has demonstrated that mixed emotions are a robust and non-artifactual experience. The evidence provided here shows that the average effect size for the elicitation of mixed emotions is medium-to-large in magnitude, and that the effect is similar across different types of mixed emotions. Second, this meta-analysis has contributed to the integration of the field of mixed emotions by demonstrating that mixed emotions have been elicited when conceptualized as dimensions or as discrete entities. Third, the effect size for mixed emotions was shown to be sensitive to the type of measure used (it is smaller for minimum index measures, and larger for direct measures) and to the gender makeup of the sample (larger for women) but not to the type of induction procedure used. Fourth, the results for the limited set of studies using subjective measures permit speculation that mixed emotions experience might be more than the sum of its constituent emotions, suggesting the importance of further investigating the subjective experience of mixed emotions. Overall, this meta-analysis provides a foundation for conducting further research on the nature, causes, and effects of mixed emotions, and to develop a more comprehensive theory of emotional complexity, which will refine our understanding of the emotional palette.

### Conflict of interest statement

The authors declare that the research was conducted in the absence of any commercial or financial relationships that could be construed as a potential conflict of interest.
